# Evaluation of away-from-home excursion patterns after falling among individuals with glaucoma: a longitudinal study

**DOI:** 10.1186/s12877-022-02788-z

**Published:** 2022-02-04

**Authors:** Catalina Garzon, Aleksandra Mihailovic, E. Jian-Yu, Sheila K. West, Laura N. Gitlin, David S. Friedman, Pradeep Y. Ramulu

**Affiliations:** 1 Wilmer Eye Institute, Johns Hopkins University School of Medicine, 600 North Wolfe Street, Maumenee B110, Baltimore, MD 21287 USA; 2 College of Nursing and Health Professions, Drexel University, Philadelphia, PA USA; 3 Massachusetts Eye and Ear, Harvard Medical School, Boston, MA USA

**Keywords:** Mobility, Physical activity, Fall, Trips, Glaucoma, Vision impairment

## Abstract

**Background:**

Unintentional falls among older adults are associated with an ensuing decline in physical activity. Our objective is to evaluate the associations between fall status and changes in excursions after a fall.

**Methods:**

Prospective cohort study of older adults with glaucoma or suspected glaucoma who reported falls for 1 year and wore a GPS device for 1-week at the baseline and 1 year later. GPS data were quantified into average: daily excursions, daily time away from home, and time per excursion. Fall status was categorized as fallers, injurious fallers, recurrent fallers, and recurrent injurious fallers. Multivariable negative binomial regression and generalized estimating equations models were employed to evaluate relationship between excursion parameters and fall status.

**Results:**

A total of 192 eligible participants were included in the analyses. Approximately half were males (50.5%) with a mean age of 70.1 years and one-fourth were Black (28.1%). There were no significant associations between fall status and end-of-study excursion parameters (*p* > 0.06 for all), and visual field damage did not modify these relationships (*p* > 0.07 for all). For instance, patients with multiple falls during a one-year study period did not demonstrate more daily excursions (incident rate ratio [IRR] = 1.16, 95% confidence interval [CI] = 0.85 to 1.57), longer time per excursion (IRR = 0.79, 95% CI =0.59 to 1.06), or more average daily time away (IRR = 1.05, 95% CI = 0.84 to 1.30) conducted at the end-of-the study. Excursion parameters at the final assessment were not significantly different from those at baseline (*p* > 0.09 for all) and the changes did not vary by fall status (*p* > 0.23 for all).

**Conclusions:**

Older adults with glaucoma did not modify their travel away from home after experiencing a fall. Additional research is necessary to understand how often maintenance of travel outside the home after a fall reflects proper compensation for greater fall risk or continued activity despite the risk of falling.

## Introduction

Unintentional falls occur in more than 1 out of 4 older people annually and continue to be the leading cause of fatal and nonfatal injuries in older adults [1, 2]. Fall accidents not only cause severe physical injuries [3, 4], but may precipitate a physical, social, and functional decline in up to a third of those affected increasing caregiver burden and institutionalization rates [5–9]. The older population with visual impairment from glaucoma is disproportionately affected by falls with a significant increase in fall risk [10, 11]. Individuals with bilateral glaucoma have also been associated with a higher likelihood of bumping into obstacles [12], reduced postural stability [13] and slower walking speed [14]. These factors along with changes in gait, balance, and hazard perception are hypothesized to contribute to the higher fall risk [15].

Mobility, defined as the transitions between different environments [16, 17], is recognized to be beneficial to maintain physical health [18]. In the older adults, this is often represented by the excursions away from home performed as part of the daily activities of living (i.e., shopping) or recreational purposes (i.e., sports, entertainment, social activities) [19]. It is possible that as an additional consequence of falling, people start spending more time at home since this may be perceived as a safer behavior. These behavioral changes in excursions away from home could be substantial, especially among the older adults with impairing conditions (i.e., visual impairments) who display a restricted pattern of mobility and physical activity [20, 21]. As shown in prior studies, persons with visual impairment conduct fewer daily excursions away from home and have a smaller excursion size when compared to their normally sighted counterparts, in the presence of impaired visual acuity [22, 23]. The possible downstream consequences of a fall on mobility are supported by prior evidence demonstrating that older adults with glaucoma who sustained an injurious fall showed a greater decline in the number of daily steps and active minutes when compared with non-fallers [24]. While the roles of falls on physical activity have been previously examined, changes in excursion patterns after a fall have not been explored.

This study aims to better define the relationship between falls and travel outside of home in a high-risk population (i.e., older adults with glaucoma) by answering several questions: After a year of prospective falls assessment, are those who have fallen, when compared to non-fallers, more likely to: 1) have a lower number of daily excursions? 2) spend less daily time away from home? 3) have shorter excursions? and, 4) have a relationship between falls and excursion patterns modified by the severity of their glaucoma? We also compared travel outside the home prior to and after the falls assessment period to determine if changes in excursion patterns differed in fallers as compared to non-fallers. We hypothesized that at the end of a one-year period of observation, participants who experienced a fall would have a lower number of excursions and shorter trips than non-fallers, likely restricting their mobility scope, and that fallers would constrict their travel outside the home over the study period.

## Methods

### Study design and study population

Our study was a prospective cohort (Falls in Glaucoma Study - FIGS) of individuals with glaucoma or suspected glaucoma recruited from the Johns Hopkins Wilmer Eye Institute between 2013 and 2015 who underwent visual and physical/travel assessments at baseline and again after 1 year, at the completion of the study period. Over this period participants prospectively reported falls. Eligible participants were 60 years of age or older within the study period, diagnosed with primary open-angle, primary angle closure, pseudoexfoliation, pigmentary or glaucoma suspect, capable of performing visual field testing, and resided within 60 miles radius from the Wilmer Eye Institute [25]. Exclusion criteria included: 1) vision impairment in either eye due to concomitant eye disease, 2) confinement to a bed or wheelchair, 3) any ophthalmic surgery within the last 2 months, 4) any hospitalizations in the past month 5) history of stroke or other neurological disorders causing visual field damage [26]. The study protocol was approved by Johns Hopkins Institutional Review Board, written informed consent was obtained from all patients, and all methods were conducted in accordance with the principles of the Declaration of Helsinki.

### Characterization and parameters of excursions

Participants were instructed to always wear a waist-worn GPS system tracker (QStarz, Inc., Taipei, Taiwan) except during bathing, swimming, and sleeping for 7 consecutive days as part of the baseline and one-year assessments. At least two calls were conducted to each participant during the one-week trials to improve compliance with wearing the device. The GPS devices pinpoint participants’ locations using the latitude and longitude coordinates on a minute-by-minute level. For each participant, the home margins were delineated as the 50-m radius from the location recorded between 2 AM and 4 AM as described in prior publications [26, 27]. Valid days of GPS data were defined as having at least 80% of location data recorded between 6 AM and 10 PM. A minimum of 2 valid days was required for the inclusion of a participant in the analysis. Valid excursions away from home were defined as transitions in location from home to away-from-home. Away from home excursions over the study week were summarized into three parameters: 1) average number of daily excursions, 2) average daily time away-from-home, and 3) average time per excursion, in accordance with previous publications [22, 23, 28].

### Collection of falls data and fall status classification

At baseline, participants were told that a fall is defined as unintentionally coming to rest on the ground or a lower level and provided with a visual description of falls via an instructional video [29]. Subsequently, participants received a set of monthly calendars and were instructed to record daily if they had any falls or not. Calendars were returned to study personnel via mail or email monthly. Those who did not return their calendars on time were called weekly to remind them to send their fall calendar information. Falls calendar data were collectible for up to 3 months at which point they were recorded as missing [27]. Each fall was further evaluated during a phone interview using a falls follow up questionnaire, where participants, among other things, reported whether they sustained any injury during a fall [25]. Fall status for the analyses was determined by classifying participants who fell at least once during the follow-up year as fallers, and more than once as recurrent fallers. Similarly, participants who sustained an injury during at least one fall were categorized as injurious fallers, and those with injuries during more than one fall as recurrent injurious fallers.

### Examination of visual function

Comprehensive visual examinations were conducted on all participants including visual acuity, contrast sensitivity, and visual field testing using the back-lit Early Treatment Diabetic Retinopathy Study (ETDRS) chart, Mars Letter Contrast Sensitivity and Humphrey Field Analyzer II using the SITA standard algorithm (Carl Zeiss Meditec, Carls-bad, California, USA), respectively. Participants wore their habitual glasses for distance and were instructed to read the characters in the back-lit ETDRS chart at a distance of 4-m. The number of letters read was transformed into logMAR values for analysis of their presenting visual acuity. Contrast sensitivity was assessed bilaterally using the Mars Letter Contrast Sensitivity (Mars Perceptrix Corporation, Chappaqua, NY, USA) and recorded in log units (logCS) [27]. Individual visual fields were reviewed by a glaucoma specialist (PR) to ensure their optimal validity and reliability with prior field patterns, if available. The pointwise sensitives from each eye were integrated by matching the spatially corresponding points and selecting the maximum sensitivity for each pair, which were eventually converted into the raw sensitivity values. The overall average of the raw sensitivities values was transformed back into dB to determine the mean sensitivity for the integrated visual field [26]. The Hodapp-Parrish-Anderson Criteria threshold values were converted to integrated visual field sensitivities and utilized to indicate none to mild glaucomatous damage if ≥28 dB, moderate glaucomatous damage if > 23 dB and < 28 dB, and severe glaucomatous damage if ≤23 dB.

### Evaluation of covariates

Demographic information such as age, sex, race, ethnicity, living/employment status, and educational attainment were obtained through standardized questionnaires. Physical capacity was gauged via body mass index, maximum grip strength, and maximum leg strength. Maximum grip strength was measured in the dominant hand using a Jamar Hand Dynamometer (Sammos Presto, Bolingbrook, IL, USA) three times, and the greatest value was recorded. Lower strength was measured twice in each leg at the distal femur using a microFET2 Dynamometer (Hoggan Scientific LLC, West Jordan, UT, USA) during a 5-s hip flexion. The maximal value from the four recordings was selected. Fifteen health comorbidities were collected, (i.e., arthritis, broken or fractured hip, back problems, history of heart attack, history of angina/chest pain, congestive heart failure, peripheral vascular disease, high blood pressure, diabetes, emphysema, asthma, stroke, Parkinson disease, cancer other than the skin cancer, and history of vertigo or Meniere disease). Medications were verified directly from the bottles when possible or recorded from the participants’ self-report. Participants with an intake of five or more prescribed medicines, excluding eye drops, were recategorized as having polypharmacy [15].

### Statistical analysis

Both baseline and final (at the end of the one-year period) excursion parameters were analyzed as continuous variables. Fall status was treated as binary variables that distinguished fallers from non-fallers, injurious fallers from those without an injurious fall, recurrent fallers from those with 1 or no falls, and recurrent injurious fallers from all subjects who did not experience > 1 injurious fall. We utilized multiple independent univariate and multivariable negative binomial regression models to evaluate the associations between each fall status and each excursion pattern at the end of the one-year period. Fall status (e.g., faller, recurrent faller, injurious faller, and injurious recurrent faller) served as the main exposure and excursions characteristic (e.g., average number of daily excursions, average time per excursion, and average daily time away) from the final assessment served as the main outcomes. Each multivariable model was adjusted for sex, age, race, comorbidities, and polypharmacy [27]. Excursion outcomes were also evaluated in additional models incorporating an interaction term with severity of visual field damage to assess whether the impact of fall status on excursion patterns varied across the spectrum of glaucoma severity. As participants contributed data across multiple study years, we employed generalized estimating equations (GEE) models to examine the correlations between fall status and excursions over each one-year study period for each participant while accounting for both the variability within each participant and between participants. Multivariable GEE models utilized excursions data from the baseline and one-year assessments as the main outcome, and were adjusted for sex, age, race, comorbidities, and polypharmacy which were determined at baseline. Additional GEE models incorporating an interaction term between visit number (i.e., baseline = 1, one-year assessment = 2) and fall status were executed to determine if any of the changes from baseline to the end of the study period in the three excursion parameters differed by fall status. All analyses were conducted using Stata 15.1 (StataCorp LLC, College Station, TX, USA).

## Results

### Demographics and visual parameters

One-hundred and ninety-two eligible participants on average had 5.7 ± 1.5 valid GPS study days for both the baseline and end-of-study assessments. There was an even sex distribution (male, *n* = 97, 50.5%) and a mean age of 70.1 ± 7.0 years (range = 57-89 years). White (63.0%) and Black (28.1%) races were predominant, and approximately 4.2% of the population was Hispanic. Most participants had a near-normal presenting visual acuity in the better eye and only 7.3% of the cohort had a logMAR ≥ 0.3, equivalent to ≥ 20/40. Median integrated visual field sensitivity was 28.1 (interquartile range (IQR) = 26.1, 29.7; normal value is ≥ 31 dB). Approximately 38.5 and 10.4% of the cohort were classified as having moderate and severe glaucomatous damage, respectively. Generally, the faller and non-faller groups were relatively similar apart from lower maximum grip strength for the dominant hand in the faller group (*p* = 0.03) (Table 1).

**Table 1:** Demographic, Vision, and Excursion Characteristics by Fall Status

Characteristic	Faller (≥1 fall), (*n* = 87)	Non-Faller (*n* = 105)	*p*-value
Age, mean (SD)	71.1 (7.1)	69.3 (6.7)	0.08
Male, n %	41 (47.1)	56 (53.3)	0.39
Black, n %	22 (25.3)	32 (30.5)	0.43
Living alone, n%	17 (19.5)	18 (17.1)	0.67
Employed, n%	27 (31.0)	41 (39.0)	0.25
Educational attainment, n %			0.77
High school degree or less	13 (14.9)	17 (16.3)	
Bachelor’s degree or some college	31 (35.7)	43 (41.3)	
Master’s or Doctorate degree	43 (49.4)	44 (42.4)	
Health			
Comorbid illness ***>*** 1,%	63 (72.4)	58 (55.2)	0.15
Polypharmacy, n %	31 (35.6)	28 (26.7)	0.18
Body Mass Index, (kg/m^2^) mean (SD)	27.3 (4.6)	27.2 (5.4)	0.89
Maximum Grip Strength for Dominant Hand, (kg) mean (SD)	30.2 (9.2)	33.5 (11.4)	0.03*
Maximum Leg Strength, (kg) mean (SD)	17.9 (5.5)	17.6 (6.4)	0.71
Vision			
Presenting Visual Acuity of better eye, logMAR, median (IQR)	0.04 (−0.02, 0.14)	0.06 (0, 0.18)	0.41
IVF Sensitivity (dB), median (IQR) ^a^	28.0 (25.9, 29.4)	28.1 (26.3, 29.9)	0.57
IVF Damage Categorized			
IVF ≥ 28 dB, mild damage	45 (51.7)	53 (50.5)	0.99
IVF > 23 dB and < 28 dB, moderate damage	33 (37.9)	41 (39.0)	
IVF ≤23 dB, severe damage	9 (10.3)	11 (10.5)	
Mean deviation of better eye, median (IQR)	−2.9 (−5.4, −0.9)	−2.4 (−5.2, − 0.4)	0.45
Binocular log Contrast sensitivity, logCS, median (IQR)	1.7 (1.7, 1.8)	1.7 (1.6, 1.8)	0.77
Fall Status			
Faller, n %	87 (100)	N/A	N/A
Recurrent Faller, n %	38 (43.7)		
Injurious Faller, n %	35 (40.3)		
Recurrent Injurious Faller, n %	13 (14.9)		
Excursions Away from Home Parameters after the Study Period ^b^			
Average number of daily excursions, median (IQR)	2.1 (1.4, 4.0)	2.4 (1.5, 4.3)	0.51
Average time per excursion (hours), median (IQR)	1.7 (0.8, 2.8)	1.5 (0.9, 2.6)	0.73
Average daily time away (hours), median (IQR)	3.9 (2.4, 5.4)	4.2 (2.3, 5.7)	1.00

*Note*. *SD* standard deviation, *n* number, *kg* kilograms, *m* meter, *logMAR* the logarithm of the minimum angle of resolution, *IQR* interquartile range, *IVF* integrated visual field, *dB* decibel, *logCS* logarithm of contrast sensitivity. Percentages were derived for the total of each group

^a^ IVF sensitivity has a normal reference value of 31 dB; *p-value ***≤*** 0.05

^b^ Excursions activity was collected over the period of a week

### Falls reports and characterization of the excursions

During the one-year-follow-up period, a total of 185 falls were reported with 55.2% of them being injurious. Almost half of the cohort sustained at least one fall (*n* = 87, 45.3%) and close to one out of five participants was a recurrent faller (*n* = 38, 19.7%). Roughly one-quarter of all participants had at least one injurious fall (*n* = 48, 25%), and thirteen participants reported multiple injurious falls (6.8%). At the end-of-the study assessment, participants conducted a median average number of daily excursions of 2.3 (IQR = 1.5, 4.2), spent a median of 1.5 h away from home during each excursion, and were away a median of 4.0 h from home daily (Table 1).

### Relationship between fall status and end-of-study period excursion parameters

Individuals who fell during the first study year, regardless of the frequency of falls or injury occurrence, were not found to be significantly different with regards to any travel patterns away from home at the end of the study period. All excursion characteristics, including average number of daily excursions, average time per excursion, and average daily time away from home, were not influenced by any fall status variable in univariate or multivariate models (Table 2) (*p* > 0.06 for all) except for recurrent fallers who had a lower average time per excursion, though only in univariate models (*p* = 0.05). The relationships between the remaining excursion patterns and fall status did not differ by visual field severity (*p* > 0.07 for all).

**Table 2:** Relationship Between Fall Status and Away-from-Home Excursions at the End of the Study Period (*n* = 192)

Fall Status	Average Number Of Daily Excursions		Average Time Per Excursion		Average Daily Time Away	
	Univariate models (IRR, 95% CI)	Multivariable models (IRR, 95% CI)	Univariate models (IRR, 95% CI)	Multivariable models (IRR, 95% CI)	Univariate models (IRR, 95% CI)	Multivariable models (IRR, 95% CI)
Faller	1.23 (0.97, 1.57)	1.11 (0.86, 1.44)	1.05 (0.85, 1.29)	1.09 (0.88, 1.35)	1.04 (0.88,1.25)	1.10 (0.92, 1.31)
Recurrent Faller	1.29 (0.96, 1.74)	1.16 (0.85, 1.57)	0.75 (0.56,1.00) *	0.79 (0.59, 1.06)	1.00 (0.80,1.24)	1.05 (0.84, 1.30)
Injurious Faller	1.15 (0.87, 1.52)	1.05 (0.79, 1.39)	1.00 (0.79, 1.28)	1.06 (0.83, 1.35)	1.11 (0.91,1.35)	1.16 (0.95, 1.41)
Injurious Recurrent Faller	1.26 (0.79, 2.01)	1.14 (0.71, 1.83)	0.88 (0.57, 1.37)	0.91 (0.59, 1.42)	1.06 (0.75,1.49)	1.11 (0.79, 1.55)

*Note*. Multivariable models adjusted for age, sex, race, integrated visual field, comorbidities, and polypharmacy. Each fall status was evaluated as a binary variable which distinguished fallers from non-fallers, injurious fallers from those without an injurious fall, recurrent fallers from those with no falls or a single fall, and recurrent injurious fallers from all subjects without >1 injurious fall

*IRR* incidence rate ratio, *CI* confidence interval; * *p*-value ***≤*** 0.05

### Change in excursion patterns from baseline to final assessments

There was no change in the average number of daily excursions, average time per excursion, and average daily time away at the end of the study compared to baseline assessment using the GEE univariable and multivariable models to account for clustering by the individual (*p*-value > 0.06 for all). The differences in excursion measures from the baseline to the end of the study period did not differ by fall status (*p*-value > 0.08 for all).

## Discussion

In this longitudinal study of adults aged 60 years and older diagnosed with glaucoma or suspect glaucoma, we found no significant association between any fall variable and excursion patterns at the end of the study period, regardless of the frequency of falls or injury occurrence in the prior year. Also, visual field damage severity did not modify the relationship between the fall status and excursion parameters. GEE analyses demonstrated that excursion patterns at the follow-up assessment did not change from baseline assessments, and these results did not differ by fall status. Thus, visually impaired adults who are prone to falls appear to continue their habitual excursions and may be exposing themselves to more falls when traveling in an external environment.

It is surprising that an experience of a fall was not associated with changes in the excursion patterns given that prior literature suggests a downstream effect of falls on functional ability, mobility, and independence [30–32]. In this same cohort, a longitudinal and objective examination of physical activity after an injurious fall revealed a reduction in the number of daily steps, active minutes, and moderate and vigorous minutes [24]. Another large cross-sectional study of men using self-reported data also found that single and recurrent fallers and those who were “very” or “somewhat” fearful of falling left their house less often [33]. It is possible that our results differed from the aforementioned study as our excursions were measured objectively using a GPS as opposed to self-report of excursions. Also, our cohort was well-educated and likely more resilient to change excursion patterns or better equipped to adapt to changes as it has been shown that high-functioning and well-educated men living in less deprived areas conduct more active trips [34, 35].

Multiple plausible explanations could be derived from the Selective Optimization with Compensation Model by Baltes and Baltes to rationalize why participants did not modify their excursion behavior after a fall [36, 37]. Selection, which refers to the restriction of activities, was not evident in our results, possibly reflecting that fewer substitutions to complete necessary work/chores outside the home (e.g., food deliveries, remote work) were available before the COVID-19 pandemic. Instead, participants continued to engage in their habitual excursions even after falls or injurious falls. Some may have optimized their behavior by pushing themselves to capacity to perform the typical excursions as these trips conferred indispensable direct benefits (e.g., groceries, medical appointments, social interactions, work, etc.). Other participants may have implemented compensatory mechanisms such as receiving help from a caregiver to complete usual excursions which paradoxically could represent a loss of independence. Additional strategies could have included the use of visual and mobility aids to facilitate the usual daily trips and counteract fall sequelae (i.e., fear of falling and being injured, decreased confidence in physical fitness, changes in gait and balance).

Given the varying degrees of visual field damage in the study population, it is also notable that the relationship between fall status and excursion parameters was neither associated nor modified by the severity of VF damage. Older adults with severe VI have been associated with an increased fall risk [10, 12], and a restricted pattern of excursions [22, 23]; nonetheless, the effects of falls on excursion parameters appear to be similar (i.e., equally unaffected) across the spectrum of glaucoma damage. When compared to the prior cross-sectional study demonstrating a restricted pattern of excursions [22], it is possible that our participants were more active (i.e., median average number of daily excursions of 2.3) than those in the other study (i.e., average of 8 to 9.2 weekly excursions) [22]. Additionally, when comparing the mean deviation of the better eye, our participants had less visual field damage (median = − 2.5 dB; IQR = -5.4,-0.6) than that observed in the prior study population (average = − 11.1 dB; SD = 7.9) potentially suggesting that restrictions in excursions may be seen at greater degrees of VF damage than those observed in our study. Similarly, our participants could also be experiencing a slower restriction in their excursion patterns such that the one-year follow-up may not be sufficient to reveal the changes in excursions associated with glaucomatous damage. On the other hand, the differences in occurrence of major life events that are associated with excursions (e.g., driving cessation) between both studies could be impacting the mobility results substantially. Considering that many study subjects had little if any visual damage, and visual field damage did not influence the relationship between fall status and excursions in our study, our results may extend to the older population without visual impairment, though overall generalizability of the study findings remains limited given the other exclusion criteria employed as part of the study (e.g., history of stroke, confinement to a bed or wheelchair, etc.).

While it could be beneficial to maintain one’s habitual excursions after a fall if proper compensatory changes were made to offset the risk of additional falls, we could not discern whether this occurred in our population. Lack of compensation, on the other hand, may represent a perceived sense of over-confidence or less awareness about the effect of fall status and/or vision impairment on one’s ability to maintain safety in environments away from home. Future studies should further characterize how excursion patterns are sustained after a fall and identify the strategies that may be used (or not used) to cope with unfamiliar environments and prevent falls.

When comparing the mobility changes over the one-year period, our participants did not exhibit any variations in their outings away from home even when considering their fall status. While the tendency to conduct fewer and shorter trips in distance and time with age has been reported [38, 39], our one-year follow-up period may not have been sufficient to observe such changes. It is also possible that excursions are not affected until the very advanced stages of physical or cognitive impairment when independence is at risk. Given that falls did not modify the excursion patterns over time in this study, more research is needed to identify the determinants of excursions and additional factors that contributed to away-from-home mobility.

Our findings must be interpreted in the context of the study’s limitations and strengths. Participants may have been subject to self-selection bias as they were more prone to report falls than study-eligible individuals from the recruitment site [40]. Though daily calls were conducted to improve compliance with the GPS wear, some participants may have forgotten to wear the devices or may have exhibited an atypical behavior during their 1 week-trial which would not be representative of their annual activity. Lack of information on other factors that influence excursions may have limited a more comprehensive assessment. Also, the degree of fear of falling has been shown to increase the fall risk and lead to a decline in physical activity among older adults with glaucoma, and could potentially also influence excursions patterns, and may influence excursions independent of falls themselves [41].

While travel away from home is an important aspect of mobility for older adults pursuing active and healthy aging, its alterations after a fall had not been examined until our study. To the best of our knowledge, our findings represent the first objective characterization of how life-space mobility is affected by falls among older adults. Our study analyzed data in a cross-sectional and longitudinal fashion and provided consistent results. Moreover, the objective characterization of falls and of habitual travel away from home using GPS devices allowed for collection of continuous measures not evaluated in prior studies.

In conclusion, our cohort of participants with a range of vision impairment (including some with normal or near-normal vision) did not exhibit any changes in their travel away from home patterns after falling, highlighting the essential role of excursions on the activities of daily living and wellness. Further research is warranted to characterize how excursions are conducted before and after falling in order to better distinguish whether maintenance of travel outside the home after a fall reflects proper compensation to fall risk as opposed to continued activity despite a high danger of falling.

## Data Availability

The datasets used and analyzed during the current study are available from the corresponding author on reasonable request.

## Abbreviations

VF: Visual fields
GPS: Geographical position system
FIGS: Falls in Glaucoma Study
ETDRS: Early Treatment Diabetic Retinopathy Study
GEE: Generalized estimating equations
